# Chlorhexidine Versus Iodine-Based Skin Preparation Was Not Associated with a Significant Difference in 90-Day Infection Risk After Instrumented Degenerative Cervical or Lumbar Spine Surgery: A Propensity Score-Matched Analysis

**DOI:** 10.3390/jcm15197714

**Published:** 2026-10-06

**Authors:** Firas Batrash, Fahad Jamal, Siddharth Jasti, James A. Belanger, Timothy A. Reiad, Tasif Mahmoud, Ahmed Iqbal, Artin Allahverdian, Bassel G. Diebo, Bryce A. Basques, Alan H. Daniels

**Affiliations:** Department of Orthopaedic Surgery, Warren Alpert Medical School of Brown University, 1 Kettle Point Avenue, East Providence, RI 02914, USA; fbgzf@umkc.edu (F.B.); fjkhp@umkc.edu (F.J.); sidjasti4@gmail.com (S.J.); jamesbelanger2025@gmail.com (J.A.B.); timothy_reiad@brown.edu (T.A.R.); tasif.mahmoud@downstate.edu (T.M.); adicxm@umkc.edu (A.I.); artin.allahverdian@md.cusm.edu (A.A.); dr.basseldiebo@gmail.com (B.G.D.); brycebasques@gmail.com (B.A.B.)

**Keywords:** chlorhexidine, povidone-iodine, surgical site infection, antisepsis, spinal fusion, spine surgery, propensity score matching, TriNetX

## Abstract

**Background/Objectives:** Chlorhexidine and iodine-based preparations are both widely used, and the existing literature suggests a possible advantage of chlorhexidine in clean elective surgery. Whether this extends to instrumented degenerative spine surgery is unknown. We asked whether chlorhexidine-based skin preparation reduces 90-day postoperative infection compared with iodine-based preparation after instrumented degenerative cervical or lumbar spine surgery. **Methods:** A retrospective TriNetX cohort study was performed among adults undergoing instrumented degenerative cervical or lumbar spine surgery. Patients were stratified by anatomic region and by preoperative preparation exposure: chlorhexidine-based versus iodine-based. Moreover, 1:1 propensity-score matching was performed within each anatomic stratum on demographics, Charlson Comorbidity Index (CCI), diabetes, smoking, obesity and BMI, immunosuppression, methicillin-resistant *Staphylococcus aureus* (MRSA) carrier status, and index procedure codes. The primary outcome was 90-day postoperative infection. Secondary outcomes were superficial and deep surgical site infection, MRSA infection, incision and drainage, debridement, sepsis, urinary tract infection, and pneumonia. **Results:** After matching, the cervical cohort included 3528 patients per group and the lumbar cohort 4164 per group. Postoperative infection did not differ in the cervical cohort (1.76% vs. 1.59%; risk ratio [RR] 1.11, 95% confidence interval [CI] 0.77–1.58; *p* = 0.578) or the lumbar cohort (3.17% vs. 3.29%; RR 0.96, 95% CI 0.76–1.22; *p* = 0.757). Superficial and deep infection, MRSA infection, debridement, sepsis, urinary tract infection, and pneumonia did not differ significantly in either cohort. **Conclusions:** No statistically significant difference in 90-day postoperative infection was found between chlorhexidine-based and iodine-based skin preparation after instrumented degenerative cervical or lumbar spine surgery.

## 1. Introduction

Surgical site infection (SSI) remains one of the most common surgical complications following degenerative spine surgery, occurring at a pooled incidence rate of between 1.4 and 3.1% [1,2]. This risk increases nearly 3-fold in the context of instrumented spine surgery, where pooled SSI incidence approaches 4.4% [2]. This trend can be attributed to additional risk factors such as longer surgical time, more extensive tissue exposure, and excessive retraction and paraspinal muscle injury [3].

SSIs increase the risk of further complications, extended length of stay, unplanned readmission, and non-routine discharge, rendering perioperative antisepsis essential [4]. Two commonly utilized preoperative antiseptic agents include chlorhexidine gluconate (CHG)-based preparations and iodine-based preparations (such as povidone-iodine) [5]. Though both are considered equally effective options in abdominal and cardiac surgery, several studies have demonstrated a possible advantage presented by CHG in reducing SSI risk, especially in the setting of clean surgery [6,7,8]. Regarding spine surgery specifically, the largest comparable study revealed a non-significant difference in SSI rates between the two, though these findings are yet to be established in a large, real-world cohort [5].

The current comparative literature in spine surgery is limited by institution-specific protocols, heterogeneous case mixes that do not specifically isolate instrumented elective procedures, or studies that do not quantify the clinical impacts of bacterial reduction [5,9]. This proves to be a significant gap, especially in the context of instrumentation, which is associated with increased rates of SSI, blood loss, operative time, and hardware-specific complications compared to non-instrumented procedures [2,3,10]. To address this gap, we aim to define whether the general trend in the clean and spine literature is consistent in instrumented populations, and whether the findings are consistent in both cervical and lumbar instrumented surgeries. We hypothesized that CHG-based preparation would be associated with a lower rate of 90-day postoperative infection than iodine-based preparation in both anatomic strata.

## 2. Materials and Methods

### 2.1. Study Design

We conducted a retrospective cohort study utilizing the TriNetX Research Network (TriNetX LLC, Cambridge, MA, USA) in July 2026 to evaluate the association between preoperative skin antisepsis strategy (CHG-based preparations vs. iodine-based preparations) and 90-day postoperative outcomes following instrumented degenerative cervical and lumbar spine surgery. TriNetX is a federated electronic health record (EHR) database encompassing healthcare organizations (HCOs) across the United States, providing access to longitudinal, patient-level data including diagnoses, procedures, medications, laboratory values, and demographics, mapped to standardized clinical terminologies. As the database contains only fully de-identified patient data compliant with the Health Insurance Portability and Accountability Act (HIPAA), this study does not constitute human subjects research under 45 CFR 46.102(e) and therefore did not require Institutional Review Board review.

Patients with an index instrumented degenerative cervical or lumbar procedure were eligible for inclusion. Analyses were executed in September 2026 against the current network of 187 healthcare organizations. Cohort sizes increased relative to the July 2026 query through data accrual, and all reported results reflect the re-executed analysis. The index date was restricted to at least 3 months before the query date so that a full 90-day observation window fell within the available data. TriNetX captures only encounters within contributing HCOs, so care received outside the network is not observed and continuous follow-up cannot be confirmed. This study is reported in accordance with the Strengthening the Reporting of Observational Studies in Epidemiology (STROBE) guideline for cohort studies (File S1).

### 2.2. Cohorts

Patients undergoing degenerative cervical or lumbar spine surgery were identified using Current Procedural Terminology (CPT) codes (Table S1). The index surgery date was defined as the date of instrumented degenerative spine surgery. Exclusion codes were also utilized to restrict to nonemergent, nonrevision, nontumor, noninfection, and nondeformity degenerative procedures. Lumbar and cervical cohorts were divided into two mutually exclusive groups based on preoperative skin preparation exposure (CHG-based preparations and iodine-based preparations) on the day of surgery. Exposure was ascertained from medication records carrying the RxNorm ingredient code for chlorhexidine (2358) or povidone-iodine (8611) dated to the index surgical encounter (Table S1). Alcohol-based and aqueous preparations could not be reliably distinguished. Exposure was restricted to inpatient order/administration on the index surgical date; outpatient dispensing and patient-reported medication history were excluded. Chlorhexidine or iodine records with oral, nasal, ophthalmic, otic and other specified non-topical routes were excluded.

### 2.3. Outcomes

Outcomes were assessed across a 90-day capture window. The primary outcome was postoperative infection, defined by ICD-10-CM codes for surgical site infection (T81.4), implant-associated infection (T84.6, T84.7), vertebral osteomyelitis or discitis (M46.2, M46.3, M46.4), spinal abscess (G06.1, G06.2), and site-specific cellulitis (L03.221 for cervical, L03.312 for lumbar). Secondary outcomes were superficial incisional infection (T81.41 with site-specific cellulitis), deep or organ-space infection (T84.6, T84.7, M46.2, M46.3, M46.4, G06.1, G06.2, and for the lumbar cohort T81.42 and T81.43), MRSA infection, incision and drainage or washout, debridement, sepsis, urinary tract infection (N39.0, N10, N30), and pneumonia (J12 to J18) (Table S1).

### 2.4. Statistical Analysis

All statistical analyses were performed within the TriNetX Research Network (TriNetX LLC, Cambridge, MA, USA). To control for confounding variables and yield comparable cohorts for analysis, propensity score matching was employed prior to evaluation. Matching was performed utilizing a one-to-one greedy matching algorithm with a caliper of 0.25 pooled standard deviations and included multiple imputations for any missing data. All cohorts were matched on age at index surgery, sex, race, Charlson Comorbidity Index (CCI) diagnoses, diabetes mellitus, smoking, obesity and body mass index, long-term systemic steroid and immunosuppressive use, chronic kidney disease, hypertension, chronic obstructive pulmonary disease, methicillin-resistant *Staphylococcus aureus* (MRSA) carrier status, and the index fusion and instrumentation procedure codes including add-on codes for additional levels (Table S1). Balance was assessed using standardized mean differences (SMDs), with an SMD < 0.10 considered acceptable. Relative risk (RR) and 95% confidence intervals (CIs) were calculated. Statistical significance was defined as *p* < 0.05. Outcome comparisons were performed across the two anatomic cohorts and no adjustment for multiple comparisons was applied; secondary outcomes are therefore considered hypothesis-generating.

## 3. Results

### 3.1. Cohort Characteristics

The cervical cohort identified 3862 patients who received CHG-based preparations and 8223 who received iodine-based preparations. The lumbar cohort identified 4384 CHG and 8157 iodine patients. After propensity-score matching, there were 3528 matched pairs in the cervical cohort and 4164 in the lumbar cohort. Baseline characteristics and covariates were comparable between matched groups, with standardized mean differences (SMDs) below 0.10 (Table 1). Mean age was 58.8 ± 12.6 years (CHG) vs. 58.6 ± 12.3 years (iodine) in the cervical cohort and 63.4 ± 12.0 vs. 63.3 ± 12.2 years in the lumbar cohort.

### 3.2. Primary Outcome: 90-Day Composite Postoperative Infection

In the cervical cohort, 90-day postoperative infection occurred in 62 of 3528 patients (1.76%) in the CHG group and 56 of 3528 (1.59%) in the iodine group (RR 1.11, 95% CI 0.77–1.58; *p* = 0.578). In the lumbar cohort, postoperative infection occurred in 132 of 4164 (3.17%) CHG and 137 of 4164 (3.29%) iodine patients (RR 0.96, 95% CI 0.76–1.22; *p* = 0.757). Neither difference was statistically significant (Table 2 and Table 3). Incisional infection (cervical 0.48% vs. 0.62%, *p* = 0.422; lumbar 1.13% vs. 0.84%, *p* = 0.183) and deep infection (cervical 0.88% vs. 0.57%, *p* = 0.122; lumbar 1.80% vs. 1.75%, *p* = 0.868) likewise did not differ.

### 3.3. Secondary Outcomes

MRSA infection and I&D/washout in the cervical cohort were too rare for reportable comparison (suppressed per small-cell policy) (Table 2). In the lumbar cohort, MRSA infection occurred in 19/4164 (0.46%) CHG vs. 17/4164 (0.41%) iodine patients (RR 1.12, 95% CI 0.58–2.15; *p* = 0.738). Rates of I&D/washout also did not differ in the lumbar cohort (34/4164, 0.82% vs. 45/4164, 1.08%; RR 0.76, 95% CI 0.49–1.18; *p* = 0.258), while debridement did not differ in either cohort (cervical 0.48% vs. 0.71%, *p* = 0.216; lumbar 0.84% vs. 1.03%, *p* = 0.363) (Table 3). In both cohorts, non-surgical site-related infectious burden did not differ in the assessed sepsis, urinary tract infection, or pneumonia outcomes (all *p* > 0.05; Table 2 and Table 3).

## 4. Discussion

This propensity-matched, national database study found no statistically significant difference in 90-day postoperative infection between CHG-based and iodine-based skin preparation, in either the cervical (1.76% vs. 1.59%, RR 1.11) or lumbar (3.17% vs. 3.29%, RR 0.96) groups. Superficial infection, deep infection, MRSA infection, debridement, sepsis, urinary tract infection, and pneumonia also did not differ in either cohort.

Degenerative spine procedures are clean (class I) wounds, so the antiseptic literature in clean surgery is the most relevant benchmark, though the value of wound classification in orthopedics has been questioned [11]. The largest recent randomized trial found povidone-iodine in alcohol noninferior to CHG in alcohol after cardiac and abdominal surgery (5.1% vs. 5.5%), whereas pooled analyses have favored CHG (RR 0.65) [6,7]. In clean surgery, the CHG benefit was confined to superficial incisional SSI, with no reduction in deep or organ/space infection, which are the categories that dominate our composite. Also, much of the pooled data compares alcohol-based CHG with aqueous iodine, so the advantage may reflect the alcohol carrier rather than the agent [8].

Spine-specific data is limited. In a prospective analysis of 6959 patients, SSI did not differ between CHG in isopropyl alcohol and aqueous povidone-iodine, even though the alcohol carrier was present only in the CHG arm, the setup most favorable to CHG [5]. Microbiological studies are mixed, with one lumbar trial finding CHG and iodine equally effective at eradicating skin flora and a meta-analysis finding fewer positive cultures with CHG [12,13]. Overall, the CHG advantage seen when clean or orthopedic cases are pooled may not carry over to instrumented spine surgery.

The absence of a statistical difference may reflect the composition of these preparations as much as the active agent. When compared in standardized alcohol-based formulations, CHG and povidone-iodine have shown similar SSI rates [6]. The three-armed NEWSkin Prep trial found iodine in alcohol noninferior to CHG in alcohol but not superior to aqueous iodine, suggesting that neither the agent nor the alcohol carrier alone explains the result and that application technique and dry time may also matter [14]. These preparations were not reliably coded within this database, and therefore, not assessed as independent covariates.

Absent a specific contraindication (e.g., allergy), either agent is a reasonable choice before instrumented degenerative spine surgery. Relevant contraindications include chlorhexidine hypersensitivity, iodine contact dermatitis, and iodine-sensitive thyroid disease [15,16,17]. These findings also suggest that prevention effort is better spent on modifiable factors such as glycemic control, obesity, smoking, anemia, and antibiotic prophylaxis timing [18,19].

### Limitations

This study has several limitations. Our analysis is retrospective and observational, so despite propensity score matching by demographics and comorbidities, unmeasured confounding by indication remains, and causality cannot be established. Additionally, skin preparation exposure relies on procedure codes and documentation within TriNetX. Complete documentation of intraoperative antiseptic usage and application may not have been coded or could have been subject to miscoding. More specifically, exposure was ascertained from RxNorm ingredient codes recorded on the day of surgery, which identify the antiseptic agent but not the setting of its use and many centers do not chart field preparation as a medication at all. In addition, intrawound vancomycin powder and other local antibiotic measures could not be controlled, because they are inconsistently coded as medications and cannot be reliably separated from systemic prophylaxis. We were also unable to adjust for HCO, so site-specific usage patterns may have driven some additional uncontrolled confounding. This concern is amplified by the modest cohort sizes relative to the size of the network, which indicate that only a small and probably non-random subset of contributing organizations codes skin preparation agents at all; the resulting population may not be representative of United States practice. Cervical MRSA and I&D outcomes were suppressed under small-cell policy, and the 90-day window may miss late deep or implant-related infections. Finally, no correction for multiple comparisons was applied. Prospective registries that record the specific product, formulation, application technique, and drying time, that capture intrawound antibiotic use and site-level protocols, and that adjudicate the source of sepsis would address these gaps directly, although resources to do so may be better invested towards addressing more modifiable infectious risk factors.

## 5. Conclusions

In patients undergoing instrumented degenerative cervical or lumbar spine surgery, CHG-based skin preparation was not associated with a statistically significant difference in 90-day postoperative infection compared with iodine-based preparation. The study was not designed to establish equivalence, and the confidence intervals do not exclude clinically relevant differences. These findings are consistent with prior reports and support directing prevention effort toward other modifiable risk factors.

## Figures and Tables

**Table 1:** Selected post-match baseline characteristics for each surgical region (cervical and lumbar) within chlorhexidine and iodine-based surgical prep cohorts.

Characteristic	Cervical CHG	Cervical Iodine	SMD	Lumbar CHG	Lumbar Iodine	SMD
Matched patients, n	3528	3528	—	4164	4164	—
Age, mean ± SD, years	58.8 ± 12.6	58.6 ± 12.3	0.018	63.4 ± 12.0	63.3 ± 12.2	0.008
Female, n (%)	1567 (44.4)	1588 (45.0)	0.012	1984 (47.6)	1962 (47.1)	0.011
White, n (%)	2795 (79.2)	2728 (77.3)	0.046	3316 (79.6)	3319 (79.7)	0.002
Black or African American, n (%)	381 (10.8)	432 (12.2)	0.045	385 (9.2)	395 (9.5)	0.008
BMI, mean ± SD	30.2 ± 6.5	30.3 ± 6.5	0.028	30.1 ± 6.1	30.2 ± 6.1	0.015
Hemoglobin A1c, mean ± SD, %	6.18 ± 1.27	6.14 ± 1.24	0.029	6.12 ± 1.03	6.10 ± 1.11	0.021
Nicotine dependence, cigarettes, n (%)	617 (17.5)	623 (17.7)	0.005	552 (13.3)	546 (13.1)	0.004
Hypertension, n (%)	2111 (59.8)	2120 (60.1)	0.005	2780 (66.8)	2767 (66.5)	0.007
Diabetes mellitus, n (%)	979 (27.7)	991 (28.1)	0.008	1220 (29.3)	1240 (29.8)	0.011
Overweight/obesity, n (%)	1187 (33.6)	1209 (34.3)	0.013	1606 (38.6)	1583 (38.0)	0.011
Chronic kidney disease, n (%)	347 (9.8)	361 (10.2)	0.013	540 (13.0)	559 (13.4)	0.014
COPD, n (%)	365 (10.3)	354 (10.0)	0.010	401 (9.6)	417 (10.0)	0.013
Systemic steroid use, n (%)	98 (2.8)	96 (2.7)	0.004	148 (3.6)	159 (3.8)	0.014
MRSA carrier/suspected carrier, n (%)	25 (0.7)	28 (0.8)	0.010	22 (0.5)	23 (0.6)	0.003

Abbreviations: CHG, chlorhexidine gluconate; COPD, chronic obstructive pulmonary disease; MRSA, methicillin-resistant *Staphylococcus aureus*; SD, standard deviation; SMD, standardized mean difference. Balance target SMD < 0.10.

**Table 2:** Ninety-day outcomes for instrumented cervical spine surgery after propensity-score matching.

Outcome	CHG n/N (%)	Iodine n/N (%)	Risk Ratio (95% CI)	*p* Value
Postoperative infection	62/3528 (1.76)	56/3528 (1.59)	1.11 (0.77–1.58)	0.578
Incisional infection	17/3528 (0.48)	22/3528 (0.62)	0.77 (0.41–1.45)	0.422
Deep infection	31/3528 (0.88)	20/3528 (0.57)	1.55 (0.89–2.71)	0.122
MRSA infection	Suppressed ^a^	Suppressed ^a^	—	—
I&D/washout	Suppressed ^a^	Suppressed ^a^	—	—
Debridement	17/3528 (0.48)	25/3528 (0.71)	0.68 (0.37–1.26)	0.216
Sepsis	44/3528 (1.25)	31/3528 (0.88)	1.42 (0.90–2.24)	0.131
Urinary tract infection	69/3528 (1.96)	57/3528 (1.62)	1.21 (0.86–1.71)	0.281
Pneumonia	46/3528 (1.30)	35/3528 (0.99)	1.31 (0.85–2.03)	0.219

Abbreviations: CHG, chlorhexidine gluconate; CI, confidence interval; I&D, incision and drainage; MRSA, methicillin-resistant *Staphylococcus aureus*. ^a^ TriNetX suppresses cell counts below 10; these outcomes could not be reported or compared.

**Table 3:** Ninety-day outcomes for instrumented lumbar spine surgery after propensity-score matching.

Outcome	CHG n/N (%)	Iodine n/N (%)	Risk Ratio (95% CI)	*p* Value
Postoperative infection	132/4164 (3.17)	137/4164 (3.29)	0.96 (0.76–1.22)	0.757
Incisional infection	47/4164 (1.13)	35/4164 (0.84)	1.34 (0.87–2.08)	0.183
Deep infection	75/4164 (1.80)	73/4164 (1.75)	1.03 (0.75–1.41)	0.868
MRSA infection	19/4164 (0.46)	17/4164 (0.41)	1.12 (0.58–2.15)	0.738
I&D/washout	34/4164 (0.82)	45/4164 (1.08)	0.76 (0.49–1.18)	0.258
Debridement	35/4164 (0.84)	43/4164 (1.03)	0.81 (0.52–1.27)	0.363
Sepsis	84/4164 (2.02)	78/4164 (1.87)	1.08 (0.79–1.46)	0.634
Urinary tract infection	174/4164 (4.18)	172/4164 (4.13)	1.01 (0.82–1.24)	0.913
Pneumonia	83/4164 (1.99)	67/4164 (1.61)	1.24 (0.90–1.70)	0.187

Abbreviations: CHG, chlorhexidine gluconate; CI, confidence interval; I&D, incision and drainage; MRSA, methicillin-resistant *Staphylococcus aureus*. Risk ratios use iodine as the reference.

## Data Availability

The data underlying this study were obtained from the TriNetX Research Network. Access to TriNetX is restricted to institutions holding an active subscription and an executed data-use agreement, and the authors are not permitted to redistribute patient-level data. The complete diagnosis, procedure, and medication code sets used to construct the cohorts and to ascertain outcomes are provided in Table S1, which permits replication by any investigator with TriNetX access. Requests for data access should be directed to TriNetX LLC https://trinetx.com (accessed on 24 September 2026).

## Supplementary Materials

The following supporting information can be downloaded at: https://www.mdpi.com/article/10.3390/jcm15197714/s1, Table S1: Diagnosis, procedure, and medication codes used for cohort construction, exclusion criteria, and outcome ascertainment; File S1: STROBE checklist for cohort studies.

## Abbreviations

The following abbreviations are used in this manuscript:

CCI	Charlson Comorbidity Index
CHG	chlorhexidine gluconate
CI	confidence interval
COPD	chronic obstructive pulmonary disease
CPT	Current Procedural Terminology
EHR	electronic health record
HCO	healthcare organization
HIPAA	Health Insurance Portability and Accountability Act
I&D	incision and drainage
ICU	intensive care unit
MRSA	methicillin-resistant *Staphylococcus aureus*
RR	relative risk
SD	standard deviation
SMD	standardized mean difference
SSI	surgical site infection
STROBE	Strengthening the Reporting of Observational Studies in Epidemiology
