# Supplementary material for: Chlorhexidine Versus Iodine-Based Skin Preparation Was Not Associated with a Significant Difference in 90-Day Infection Risk After Instrumented Degenerative Cervical or Lumbar Spine Surgery: A Propensity Score-Matched Analysis
Source: J Clin Med. 2026 Oct 6;15(19):7714. doi: 10.3390/jcm15197714 (PMC13666177; doi:10.3390/jcm15197714)
Supplement: Supplementary file 1 [file jcm-15-07714-s001.zip › Supplementals/File S1_STROBE_RED.pdf]

STROBE Statement—checklist of items that should be included in reports of observational studies

Manuscript: Chlorhexidine versus Iodine-Based Skin Preparation Was Not Associated with a Significant Difference in 90-Day Infection Risk After Instrumented Degenerative Cervical or Lumbar Spine Surgery: A Propensity Score Matched Analysis (revised September 2026; page and line numbers refer to the revised manuscript)

| | Item No | Recommendation | Location in manuscript (section, page, line) |
|---|---|---|---|
| **Title and abstract** | 1 | (*a*) Indicate the study's design with a commonly used term in the title or the abstract | Title (p. 1, lines 2–5): "…A Propensity Score Matched Analysis"; Abstract, Methods (p. 1, lines 14–37): "retrospective TriNetX cohort study". |
| | | (*b*) Provide in the abstract an informative and balanced summary of what was done and what was found | Abstract (p. 1, lines 14–37). |
| **Introduction** | | | |
| Background/rationale | 2 | Explain the scientific background and rationale for the investigation being reported | Section 1, Introduction (pp. 1–2, lines 41–66). |
| Objectives | 3 | State specific objectives, including any prespecified hypotheses | Section 1, Introduction, final paragraph (p. 2, line 64): aim and prespecified hypothesis. |
| **Methods** | | | |
| Study design | 4 | Present key elements of study design early in the paper | Abstract, Methods (p. 1, lines 14–37); Section 2.1, Study Design (p. 2, lines 68–88). |
| Setting | 5 | Describe the setting, locations, and relevant dates, including periods of recruitment, exposure, follow-up, and data collection | Section 2.1, Study Design (p. 2, lines 68–88): TriNetX network, 187 healthcare organizations, analyses executed September 2026 (p. 2, line 81), 90-day capture window. |
| Participants | 6 | (*a*) *Cohort study*—Give the eligibility criteria, and the sources and methods of selection of participants. Describe methods of follow-up<br>*Case-control study*—Give the eligibility criteria, and the sources and methods of case ascertainment and control selection. Give the rationale for the choice of cases and controls<br>*Cross-sectional study*—Give the eligibility criteria, and the sources and methods of selection of participants | Section 2.2, Cohorts (pp. 2–3, lines 89–102); exposure definition (p. 3, line 96); Table S1. |
| | | (*b*) *Cohort study*—For matched studies, give matching criteria and number of exposed and unexposed<br>*Case-control study*—For matched studies, give matching criteria and the number of controls per case | Section 2.2, Cohorts (pp. 2–3, lines 89–102) and Section 2.4, Statistical Analysis (p. 3, lines 112–127): 1:1 propensity matching and covariate list; matched numbers in Section 3.1 (pp. 3–4, lines 129–8) and Table 1 (p. 4, line 145). |
| Variables | 7 | Clearly define all outcomes, exposures, predictors, potential confounders, and effect modifiers. Give diagnostic criteria, if applicable | Section 2.2, Cohorts (pp. 2–3, lines 89–102): exposure; Section 2.3, Outcomes (p. 3, lines 103–111): primary and secondary outcomes with codes; Table S1. |

| | | | |
|---|---|---|---|
| Data sources/ measurement | 8* | For each variable of interest, give sources of data and details of methods of assessment (measurement). Describe comparability of assessment methods if there is more than one group | Sections 2.1 through 2.4 (p. 2, lines 68–88 to p. 3, lines 112–127); Table S1 gives all code sets. |
| Bias | 9 | Describe any efforts to address potential sources of bias | Section 2.2, Cohorts (pp. 2–3, lines 89–102): exposure restricted to inpatient index-day records with route exclusions; Section 2.4 (p. 3, lines 112–127): propensity matching, SMD threshold, no multiplicity correction (Section 2.4). |
| Study size | 10 | Explain how the study size was arrived at | Section 3.1, Cohort Characteristics (pp. 3–4, lines 129–8): pre- and post-match counts; no a priori sample size calculation, all eligible network patients were included. |
| Quantitative variables | 11 | Explain how quantitative variables were handled in the analyses. If applicable, describe which groupings were chosen and why | Section 2.4, Statistical Analysis (p. 3, lines 112–127); Table 1 (p. 4, line 145): age, BMI, and A1c as mean ± SD |
| Statistical methods | 12 | (*a*) Describe all statistical methods, including those used to control for confounding | Section 2.4, Statistical Analysis (p. 3, lines 112–127). |
| | | (*b*) Describe any methods used to examine subgroups and interactions | Sections 2.1 and 2.2 (p. 2, lines 68–88; pp. 2–3, lines 89–102): analyses stratified by anatomic region; no interaction tests. |
| | | (*c*) Explain how missing data were addressed | Section 2.4 (p. 3, line 117): multiple imputation within the TriNetX matching procedure. |
| | | (*d*) *Cohort study*—If applicable, explain how loss to follow-up was addressed<br>*Case-control study*—If applicable, explain how matching of cases and controls was addressed<br>*Cross-sectional study*—If applicable, describe analytical methods taking account of sampling strategy | Section 2.1 (p. 2, line 85): follow-up limited to in-network encounters; Section 3.1 (Section 3.1); Section 4.1, Limitations (p. 6, lines 200–221). |
| | | (*e*) Describe any sensitivity analyses | Section 2.3 (Section 2.3): infection-only primary outcome adopted at review, incisional and deep infection reported separately; Section 2.4 (Section 2.4): eighteen uncorrected comparisons treated as hypothesis-generating. |

**Results**

| | | | |
|---|---|---|---|
| Participants | 13* | (a) Report numbers of individuals at each stage of study—eg numbers potentially eligible, examined for eligibility, confirmed eligible, included in the study, completing follow-up, and analysed | Section 3.1, Cohort Characteristics (p. 3, lines 122–127): numbers identified and numbers matched for both cohorts. Stage-by-stage exclusion counts are not exposed by the TriNetX interface. |
| | | (b) Give reasons for non-participation at each stage | Section 2.2, Cohorts (pp. 2–3, lines 88–100); Table S1: all exclusion criteria and their codes are listed. The number excluded at each stage is not available from the platform. |

| | | | |
|---|---|---|---|
| | | (c) Consider use of a flow diagram | Not applicable: the TriNetX platform does not generate a participant flow diagram. |
| Descriptive data | 14* | (a) Give characteristics of study participants (eg demographic, clinical, social) and information on exposures and potential confounders | Section 3.1, Cohort Characteristics (p. 3, lines 121–130); Table 1 (p. 4, lines 138–142): demographics, comorbidities, and standardized mean differences for both matched cohorts. |
| | | (b) Indicate number of participants with missing data for each variable of interest | Not applicable: TriNetX returns cohort counts after multiple imputation; per-variable missingness is not exposed by the platform. |
| | | (c) *Cohort study*—Summarise follow-up time (eg, average and total amount) | Section 2.3, Outcomes (p. 3, line 102): a fixed 90-day capture window was applied; mean observed follow-up time is not summarized by the platform. |
| Outcome data | 15* | *Cohort study*—Report numbers of outcome events or summary measures over time | Section 3.2, Primary Outcome (pp. 3–4, lines 131–137); Section 3.3, Secondary Outcomes (p. 5, lines 153–167); Tables 2 and 3 (p. 4, lines 143–152): event counts and denominators for every outcome in both cohorts. |
| | | *Case-control study*—Report numbers in each exposure category, or summary measures of exposure | Not applicable: no unadjusted (pre-match) outcome comparisons are reported; all estimates are post-match. |
| | | *Cross-sectional study*—Report numbers of outcome events or summary measures | Not applicable: no person-time denominators; a fixed 90-day window was used for all patients. |
| Main results | 16 | (*a*) Give unadjusted estimates and, if applicable, confounder-adjusted estimates and their precision (eg, 95% confidence interval). Make clear which confounders were adjusted for and why they were included | Section 2.4, Statistical Analysis (p. 3, lines 110–115): matching covariates; Section 3.2 (pp. 3–4, lines 131–137) and Section 3.3 (p. 5, lines 153–167); Tables 2 and 3 (p. 4, lines 143–152): risk ratios with 95% confidence intervals, iodine as the reference group. Propensity-score matching is the confounding adjustment; unmatched estimates are not reported. |
| | | (*b*) Report category boundaries when continuous variables were categorized | Not applicable: no continuous exposure variables were categorized. |
| | | (*c*) If relevant, consider translating estimates of relative risk into absolute risk for a meaningful time period | Section 4, Discussion (p. 6, lines 217–218): absolute risk difference of 1.2% reported for the lumbar sepsis comparison. Relative risks are reported throughout Tables 2 and 3. |
| Other analyses | 17 | Report other analyses done—eg analyses of subgroups and interactions, and sensitivity analyses | Section 3.3, Secondary Outcomes (p. 5, lines 153–167); Tables 2 and 3 (p. 4, lines 143–152): subgroup analysis by anatomic region (cervical, lumbar). No sensitivity analyses were performed. |
| **Discussion** | | | |
| Key results | 18 | Summarise key results with reference to study objectives | Section 4, Discussion, opening paragraph (p. 5, lines 169–175); Section 5, Conclusions (p. 7, lines 255–262). |
| Limitations | 19 | Discuss limitations of the study, taking into account sources of potential bias or imprecision. Discuss both direction and magnitude of any potential bias | Section 4, Discussion (p. 6, lines 210–223): interpretation of the isolated lumbar sepsis signal, including the multiple-comparisons argument at lines 218–220; Section 4.1, Limitations (pp. 6–7, lines 232–253). |

| | | | |
|---|---|---|---|
| Interpretation | 20 | Give a cautious overall interpretation of results considering objectives, limitations, multiplicity of analyses, results from similar studies, and other relevant evidence | Section 4, Discussion (pp. 5–6, lines 169–230); Section 5, Conclusions (p. 7, lines 255–262). |
| Generalisability | 21 | Discuss the generalisability (external validity) of the study results | Section 2.1, Study Design (p. 2, lines 71–77): United States multi-institutional network; Section 4.1, Limitations (p. 6, lines 244–247): generalisability addressed directly, noting that only a small and probably non-random subset of contributing organizations codes skin preparation agents, so the population may not represent United States practice. |
| **Other information** | | | |
| Funding | 22 | Give the source of funding and the role of the funders for the present study and, if applicable, for the original study on which the present article is based | Funding (p. 7, line 272): "This research received no external funding." The role of the funder is addressed in Conflicts of Interest (p. 7, lines 292–295). |

*Give information separately for cases and controls in case-control studies and, if applicable, for exposed and unexposed groups in cohort and cross-sectional studies.

**Note:** An Explanation and Elaboration article discusses each checklist item and gives methodological background and published examples of transparent reporting. The STROBE checklist is best used in conjunction with this article (freely available on the Web sites of PLoS Medicine at http://www.plosmedicine.org/, Annals of Internal Medicine at http://www.annals.org/, and Epidemiology at http://www.epidem.com/). Information on the STROBE Initiative is available at www.strobe-statement.org.
