# Supplementary material for: Chlorhexidine Versus Iodine-Based Skin Preparation Was Not Associated with a Significant Difference in 90-Day Infection Risk After Instrumented Degenerative Cervical or Lumbar Spine Surgery: A Propensity Score-Matched Analysis
Source: J Clin Med. 2026 Oct 6;15(19):7714. doi: 10.3390/jcm15197714 (PMC13666177; doi:10.3390/jcm15197714)
Supplement: Supplementary file 1 [file jcm-15-07714-s001.zip › Supplementals/Table S1_R1_RED.pdf]

Table S1. Diagnosis, procedure, and medication codes used for cohort construction, exclusion criteria, and outcome ascertainment.

Concept	Codes
**Cohort definitions**	
Chlorhexidine exposure (same-day skin preparation)	RxNorm-2358; restricted to inpatient administration or inpatient order on the index date; outpatient dispensing and reported history excluded; oral, nasal, ophthalmic, otic, vaginal, inhalational, injectable, and implant routes excluded
Povidone-iodine exposure (same-day skin preparation)	RxNorm-8611; restricted to inpatient administration or inpatient order on the index date; outpatient dispensing and reported history excluded; oral, nasal, ophthalmic, otic, vaginal, inhalational, injectable, and implant routes excluded
Cervical fusion anchor	CPT-22551, CPT-22554, CPT-22590, CPT-22595, CPT-22600
Cervical implant/instrumentation requirement	CPT-22840, CPT-22842, CPT-22845, CPT-22853, CPT-22854, CPT-22856
Lumbar fusion anchor	CPT-22558, CPT-22585, CPT-22612, CPT-22614, CPT-22630, CPT-22632, CPT-22633, CPT-22634
Lumbar implant/instrumentation requirement	CPT-22840, CPT-22841, CPT-22842, CPT-22845, CPT-22853, CPT-22854, CPT-22859
**Exclusion criteria**	
Prior MRSA screening result (used to define carrier status for matching)	LOINC-35492-8, LOINC-52969-3
Prior/revision spine procedure	CPT-22830, CPT-22849, CPT-22850, CPT-22852, CPT-22855
Trauma/fracture	ICD10CM-S12, ICD10CM-S22, ICD10CM-S32
Active/prior infection	ICD10CM-T84.5, ICD10CM-T84.6, ICD10CM-T84.7, ICD10CM-M46.2, ICD10CM-M46.3, ICD10CM-L03, ICD10CM-G06.1, ICD10CM-G06.2, ICD10CM-A18.0
Deformity/complex reconstruction	CPT-22843, CPT-22844, CPT-22846, CPT-22847, CPT-22848, CPT-22206, CPT-22207, CPT-22208, CPT-22210, CPT-22212, CPT-22214, CPT-22216, CPT-22220, CPT-22222, CPT-22224, CPT-22226, ICD10CM-M40, ICD10CM-M41, ICD10CM-Q67.5, ICD10CM-Q76.3, ICD10CM-Q76.42
Tumor/oncology	ICD10CM-C40–C41, ICD10CM-C79.51, ICD10CM-C79.52, ICD10CM-D48.0, ICD10CM-D49.2
**Outcomes**	
Postoperative infection (primary outcome)	ICD10CM-T81.4, ICD10CM-T84.6, ICD10CM-T84.7, ICD10CM-M46.2, ICD10CM-M46.3, ICD10CM-M46.4, ICD10CM-G06.1, ICD10CM-G06.2, ICD10CM-L03.221 (cervical cohort), ICD10CM-L03.312 (lumbar cohort)
Superficial infection	ICD10CM-T81.41, ICD10CM-L03.221 (cervical), ICD10CM-L03.312 (lumbar)

Deep infection	ICD10CM-T84.6, ICD10CM-T84.7, ICD10CM-M46.2, ICD10CM-M46.3, ICD10CM-M46.4, ICD10CM-G06.1, ICD10CM-G06.2; lumbar cohort additionally ICD10CM-T81.42, ICD10CM-T81.43
MRSA infection	ICD10CM-B95.62, ICD10CM-A41.02, ICD10CM-A49.02, ICD10CM-J15.212, LOINC-13317-3
I&D/washout	CPT-10060, CPT-10061, CPT-22015
Debridement	CPT-11042, CPT-11043, CPT-11044, CPT-11045, CPT-11046, CPT-11047
Sepsis	ICD10CM-A41, ICD10CM-R65.2, ICD10CM-T81.44, ICD10CM-A40
Urinary tract infection	ICD10CM-N39.0, ICD10CM-N10, ICD10CM-N30
Pneumonia	ICD10CM-J12, ICD10CM-J13, ICD10CM-J14, ICD10CM-J15, ICD10CM-J16, ICD10CM-J17, ICD10CM-J18

Abbreviations: CPT, Current Procedural Terminology; I&D, incision and drainage; ICD10CM, International Classification of Diseases, 10th Revision, Clinical Modification; LOINC, Logical Observation Identifiers Names and Codes; MRSA, methicillin-resistant *Staphylococcus aureus*; RxNorm, normalized naming system for clinical drugs.
